# A Surgical Hazard During Adenoidectomy in Children—A Case Report

**DOI:** 10.3390/children12020139

**Published:** 2025-01-27

**Authors:** Željka Roje, Zlatko Kljajić, Krešimir Kolić, Darko Ilić

**Affiliations:** 1 Private Practice of Otorhinolaryngology, 21 000 Split, Croatia; droje@mefst.hr; 2 Faculty of Maritime Studies, University of Split, R. Boskovica 37, 21 000 Split, Croatia; 3 Clinical Department of Diagnostic and Interventional Radiology, University Hospital Center Split, 21 000 Split, Croatia; kk@mefst.hr; 4 Department of Anesthesiology and Intensive Care, University Hospital Split, 21 000 Split, Croatia; poliklinika@priska-med.com

**Keywords:** internal carotid artery, tonsillectomy and adenoidectomy, surgery, pharynx

## Abstract

**Background:** Adenoidectomy is one of the world’s most frequently performed surgical procedures. Although the operation is relatively simple and with a very low percentage of complications, it can sometimes be accompanied by fatal complications. One such scenario arises when an unrecognized aberrant course of the internal carotid artery is present, particularly if the artery is near or in contact with the oropharynx or nasopharynx. **Methods:** This report presents the case of a 3-year-old girl scheduled for adenoidectomy and the placement of ear aeration tubes. Following intubation, the oro-nasopharynx was inspected during the procedure, revealing a pulsatile mass suspected to be an aberrant right internal carotid artery (ICA). **Results:** A contrast-enhanced MSCT of the neck was performed, confirming the presence of an aberrant right carotid artery in direct contact with the posterior wall of the nasopharynx. **Conclusions:** In conclusion, a thorough visual and palpable examination by an otorhinolaryngologist after the induction of anesthesia, with the child’s neck in extension, is crucial for identifying aberrant carotid artery courses. Such careful assessment can help prevent potentially fatal complications.

## 1. Introduction

Adenoidectomy is one of the most commonly performed pediatric surgical procedures worldwide. Although the evidence in the literature remains limited, recurrent upper respiratory infections, otitis media with effusion, and obstructive sleep apnea syndrome are widely regarded as the primary indications for adenoidectomy [1]. The surgical procedure is relatively simple and safe, with few complications. However, potentially life-threatening hemorrhaging can occur during routine nasopharyngeal surgery due to nasopharyngeal internal carotid artery (ICA) aberrancy. In a retrospective cohort study of 504,262 children undergoing tonsillectomy, the overall rate of postoperative death was 7 per 100,000 operations [2]. However, while the mortality rate for adenoidectomy is lower, the operative risk remains significant if an anatomical aberration of the internal carotid artery is not identified in time. Otolaryngologists need to perform thorough visual and physical examinations before proceeding with surgery to minimize the risk of severe or fatal hemorrhage [3].

The common extracranial part of the internal carotid artery (ICA) usually ascends straight in the parapharyngeal space to the base of the skull, without branching [4]. The normal anatomical path of the cervical internal carotid artery is posterolateral to the lateral pharyngeal wall, with an age-dependent distance from the tonsillar fossa approaching 2.5 cm in adults. This positioning protects against iatrogenic injury during tonsillectomy and adenoidectomy [5]. However, variations in the course and position of the cervical internal carotid arteries (ICAs) are well documented and extensively described in both the radiology and otorhinolaryngology literature [6]. Pronounced parapharyngeal ICA aberrations are found in up to 5% of the general population and can place the vessel near the pharyngeal wall, where it is at increased risk of injury, particularly during surgical procedures [7]. Weibel and Fields, who distinguished between tortuosity, kinking, and coiling of the vessel [8], first described anatomical variations in the ICA in 1965. The problem with the anatomical classification of aberrant ICAs is that it is not particularly useful to clinicians, as it does not correlate with the risk of ICA injury. In 2008, Pfeifer and Ridder proposed a clinical–radiological classification of parapharyngeal ICA aberrations, considering the risk of potential ICA injury, the location within the naso-, oro-, or hypopharynx, and the minimum distance to the pharyngeal wall [9]. According to this classification, ICA variations may reduce the distance to less than 1 mm. This distance is age- and weight-dependent in children and is generally smaller. Since surgical procedures in the throat, such as tonsillectomies and adenoidectomies, are most commonly performed in children and adolescents, an aberrant ICA poses an even higher risk of incidental injury in this patient group [10]. One of the problems in recognizing the aberrant course of the ICA is that most patients are asymptomatic [11]. An aberrant course of the ICA can present as a submucosal pseudomass upon direct inspection of the pharynx, posing a significant potential hazard during pharyngeal surgery or biopsy [12]. When symptoms are present, dysphagia, dysphonia, cervical bolus sensation, and glossopharyngeal neuralgia have been reported [9]. In this article, we present the case of a 3-year-old girl in whom an ICA aberration was detected after general anesthesia, just before the surgical procedure. The aberrant right internal carotid artery was confirmed through radiological examinations, thereby avoiding a potential surgical hazard.

## 2. Case Report

A 3-year-old girl, accompanied by her parents, presented for an otorhinolaryngological examination, complaining of recurrent acute inflammation in the right ear every 15 days. The parents of the child have given consent for their child’s data to be used for the purposes of the case report and the respective publication. On one occasion, thick, colored discharge was noted from the right ear. Each episode of right ear infection was accompanied by a fever over 38 °C, nasal congestion, and rhinorrhea. The left ear had not experienced similar issues. Her medical history revealed occasional snoring but no signs of apnea.

Apart from the current illness and a previous episode of acute purulent tonsillitis, there were no other notable medical conditions. No family members had a history of hearing problems. On ENT examination, the patient was alert, in good general condition, eupneic, afebrile, and notably pale. Oropharyngoscopy revealed purulent secretion on the posterior pharyngeal wall and grade 2-tonsil hypertrophy. Anterior rhinoscopy showed a small amount of purulent secretion in both nasal cavities. Otoscopic examination revealed blurred eardrums, and polyadenopathy was noted in the neck.

During fiberendoscopy of the nose and nasopharynx, purulent secretion was observed in both ostiomeatal complexes, and enlarged (grade 3) adenoid vegetations covered with purulent secretion were seen in the epipharynx. The tympanogram showed a flat bilateral curve (B curve).

A diagnosis of bilateral secretory otitis and chronic adenoiditis was made, and according to the national guidelines, adenoidectomy and bilateral myringotomy were indicated. After the preoperative treatment, the date for the planned surgery was scheduled. On operation day, the child was intubated. The anesthetic induction proceeds without complications.

The preparation of the operative field for the planned adenoidectomy began, and after placing the Boyle–Davis opener in the oral cavity, the oropharynx was fully visualized. However, adenoidectomy was not performed because, during the preparation of the operating field, a large pulsating mass was observed in the midline of the posterior oropharyngeal wall. The surgeon suspected it could be the right internal carotid artery, as the mass originated on the right side of the oropharyngeal wall, ascending toward the epipharynx in the midline and reaching the adenoids.

**Figure 1:**
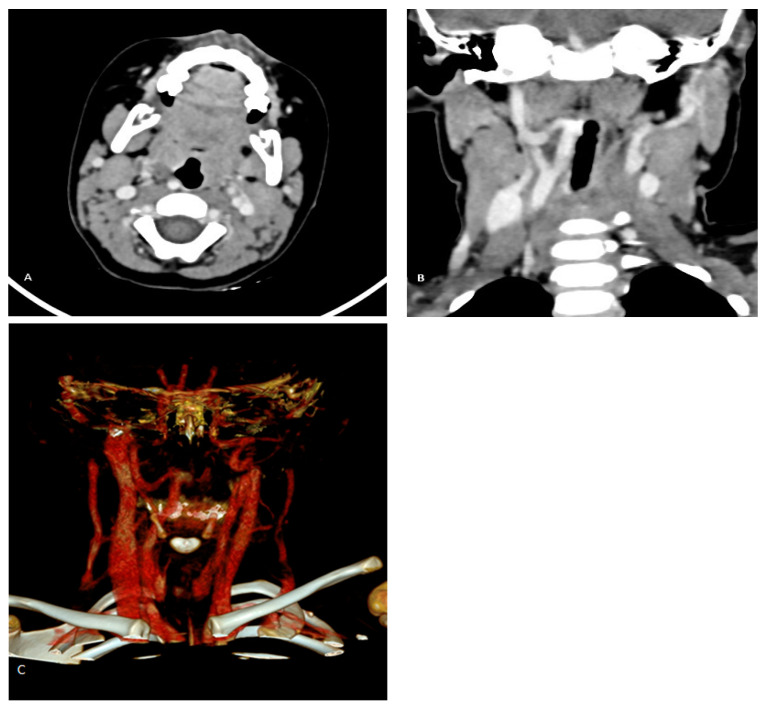
(**A**–**C**) Right-sided ICA aberration in a 3-year-old child. Axial MSCT scan shows that the right ICA courses retropharyngeally, reaching and displacing the oropharyngeal wall (**A**). Coronal MSCT reconstruction reveals right ICA tortuosity with kinking and medialization of the vessel (**B**). Volume rendering displays the course of the right ICA (**C**).

Since the risk of bleeding due to damage to a large blood vessel exceeds the planned benefit of the procedure, further procedure (adenoidectomy) was abandoned with the advice to perform a contrast-enhanced MSCT of the neck to confirm or exclude possible unusual and dangerous position of the internal carotid artery. The MSCT was performed using a 128-slice Siemens Somatom scanner (Munich, Germany). The scan revealed an anomalous medial and retropharyngeal course of the right ICA at the level of the oropharynx. The aberrant vessel was in direct contact with the pharyngeal wall, displacing it (Figure 1). Using the clinicoradiological classification system proposed by Pfeifer and Ridder [7], this case corresponded to a grade IV aberrant ICA.

## 3. Discussion

We presented the case of a 3-year-old child who was indicated for surgical treatment—adenoidectomy and placement of aeration tubes due to tissue hypertrophy of adenoid vegetations and secretory otitis. Although these procedures are generally routine in otolaryngology, complications can be serious and even fatal. Bearing in mind the anatomical relationship of the internal carotid artery to the structures of the oropharynx and nasopharynx, as part of the preoperative examination, the surgeon must consider the possibility of an aberrant course of the internal carotid artery to recognize the risk of surgical damage with possibly fatal consequences in time.

In [13], it has to be pointed out that the following abnormality causes, like embryological maldevelopment and cardiovascular disease, are suspected to be the main causes of ICA abnormalities, although the etiology is unknown. Also, the embryological development of the ICA originates from the third aortic arch and the cranial part of the dorsal aorta, with loops forming at the junction of the components mentioned above during the first fetal month. Likewise, in the second month of fetal development, the artery straightens and elongates as the heart and great vessels descend into the mediastinum.

The variations in the ICA are congenital in origin, and the failure of the structures mentioned above to descend into the thorax has been identified as a potential cause for the formation of vascular loops in the pharynx, although no definitive source in the literature confirms this [14].

An anatomical study on the course of the ICA in head and neck cadaver specimens was conducted by Paulsen et al. Further, out of 282 specimens, 74 (26.2%) exhibited a curved course to the base of the skull, while 17 (6%) showed pronounced kinking or coiling [14]. Other studies, based on angiographic and cadaveric findings, have reported the incidence of these anomalies to be between 1% and 10% [13]. The presence of an anomalous vessel increases the risk of injury during routine pharyngeal procedures. Even though iatrogenic injuries of ICA abnormalities remain relatively rare, a higher incidence in specific patient groups like velocardiofacial syndrome is still at risk [15].

The normal anatomical course of the ICA is posterolateral to the pharyngeal wall, with an age-dependent distance from the tonsillar fossa approaching 2.5 cm in adults [14]. The typical position of the ICA is parallel posterior or posterolateral and vertical to the tonsillar fossa. The ICA is located anterior to the posterior pillar by a distance equal to 40% of the width of the corresponding tonsillar fossa [16]. Deutsch et al. further described the tonsillocarotid distance in the pediatric population using magnetic resonance imaging (MRI), approaching 25 mm [17]. The same study analyzed the distance between a straight-running extracranial ICA and the tonsillar fossa, which averaged 14 mm in a 1-year-old child and approached 25 mm at 12–15 years or a body weight of 56 kg. Variations in the course of the ICA may reduce this distance in a potentially dangerous manner. Clinical scientific studies have rarely addressed the significance of pulsations of the pharyngeal wall during tonsillectomies and adenoidectomies in children [17].

Weibel and Fields classified extracranial ICA aberrations [8], where the anatomical course of the ICA is effectively outlined, taking into account the vessel’s shape. This helps to evaluate whether symptoms of cerebrovascular insufficiency may result from an ICA variation. However, it does not account for the vessel’s relationship to the pharyngeal wall, making it clinically insufficient for assessing the risk of iatrogenic or accident-induced ICA injury [9].

Furthermore, the clinicoradiological classification system for transoral procedures was proposed by Pfeiffer and Ridder [9].

The clinicoradiologic grading system for extracranial internal carotid artery aberrations considers two important parameters: the vessel’s minimal distance from the pharyngeal wall and the corresponding pharyngeal level. This grading system is applicable for medialized vessel courses, with a reasonable distribution of grade I to grade IV anomalies [9]. Evidently, aberrant ICA is very important for planning transoral interventional and surgical procedures.

However, since many patients with ICAs have vessels that bend toward the surface of the neck rather than toward the pharynx, the clinicoradiologic grading system was expanded in 2015 by the same authors to include these anomalies [7].

Most ICA aberrations involve medial deviations. When the ICA deviates medially, the distances to the orifice of Rosenmüller’s fossa (RF), the posterior nasopharyngeal wall (PW), and the midline of the posterior nasopharyngeal wall (ML) are shortened [18].

Several studies have previously been conducted on parapharyngeal ICA aberrations, most of which are adult case reports. Larger case series in children have focused on this anatomical variation’s etiology and potential neurological manifestations. However, the impact of ICA aberrations on the timely recognition of these variations, specifically for preoperative and intraoperative identification, has not yet been emphasized.

The problem in detecting aberrant internal carotid arteries is that they are asymptomatic in most cases, regardless of age [5]. Symptoms such as foreign body sensation, dysphagia, or odynophagia have been attributed to ICA aberrations in older patients but have not been reported in children [19]. However, it has been previously discussed that looped and kinked extracranial carotid arteries can cause neurological disorders, even in children [20]. In a study involving nine children, ICA variations and clinical symptoms, such as hemiplegia and dizziness, were reported [21].

Concerning the asymptomatic nature of ICA, it is crucial to be familiar with the available evaluation modalities [19]. To recognize an aberrant course of the internal carotid artery, a thorough preoperative otorhinolaryngological examination is essential, focusing on inspection and palpation of the oropharynx and nasopharynx and fiberoptic endoscopy. It has to be noticed that careful palpation is very important to rule out aberrant ICA and prevent all mentioned risks [5]. Regardless, our recommendation is to perform a detailed inspection and palpation of the oropharynx and nasopharynx while the child is under anesthesia, with the neck in hyperextension, as this provides a clearer view and allows for the identification of any anatomical aberrations, such as pulsations in the area of the lateral wall of the oropharynx and nasopharynx.

Preoperative imaging is necessary if a suspicious finding is noted during the preoperative otorhinolaryngological examination that could suggest an aberration of the internal carotid artery. MSCT of the neck provides definitive confirmation of an aberrant carotid artery, while EcoColorDoppler (ECD) offers valuable information regarding the evaluation of carotid vessels [19]. Additionally, the neck’s Magnetic Resonance Imaging (MRI) provides spatial information about the adjacent pharyngeal anatomy [22]. In cases of suspected aberrant carotid arteries, some authors also recommend preoperative ultrasound of the blood vessels or MRI with angiography [10].

It is also important to note that internal carotid artery repositioning cases have been described in the scientific literature. A study involved a case of a 28-year-old African American female with no history of radiation therapy or neck surgery who underwent two neck CT scans taken 7 months apart. The first scan identified retropharyngeal ICAs at the level of the oropharynx, while the second scan, taken several months later, showed the arteries had returned to their normal (non-retropharyngeal) position [6].

The mechanism underlying the variability in the position of the internal carotid artery (ICA) in these cases remains unclear, and there were no follow-up or prior images available to determine if or when the arteries changed course in our patients. Having the potential for changes in the position of this artery over time, it is recommended to perform a scan right before planning and conducting any surgery near the pharynx [23]. However, since changes in the position of the ICA can occur within short periods, clinical correlation through thorough visual inspection and digital exploration of the pharynx is always advised [24].

When ICA aberrations in children are identified, the indication for the proposed procedure should be carefully reevaluated. The surgery should only proceed with special precautions or be canceled entirely if the risk of potential complications outweighs the expected benefit of the treatment.

## 4. Conclusions

The study presents the case of a three-year-old girl scheduled for surgery to place aeration tubes in the ear and adenoidectomy. After proceeding with intubation and anesthesia, the placement of a ventilation tube was performed. Further, during the inspection of the oro-nasopharynx, a pulsatile mass was observed on the posterior wall. The surgery was canceled due to suspicion of aberrant flow of the right internal carotid artery. MSCT of the neck was performed, and the clinical suspicion was confirmed.

Although aberrant ICA is relatively rare and major bleeding during adenoidectomy is even rarer, we strongly recommend a detailed inspection and palpation of the oro- and nasopharynx after intubation and before starting the surgical procedure. From our practice, this examination would potentially prevent fatal outcomes in patients with aberrant carotid arteries without prolonging the surgical time or placing an economic burden on the healthcare system.

Given these factors, we recommend a more practical approach: preferentially, the surgeon should inspect and palpate the oropharynx and nasopharynx in hyperextension. The inspection of the adenoid should be performed by lifting the soft palate, making visualization of the posterior pharyngeal wall possible. Ultimately, the clinician should decide to proceed with surgery in consultation with the radiologist and the child’s parents.

## Data Availability

The dataset is available upon request from the authors due to privacy, legal and ethical reasons.
